# Evasion of Classical Complement Pathway Activation on *Plasmodium falciparum*-Infected Erythrocytes Opsonized by PfEMP1-Specific IgG

**DOI:** 10.3389/fimmu.2018.03088

**Published:** 2019-01-07

**Authors:** Mads Delbo Larsen, Maria del Pilar Quintana, Sisse Bolm Ditlev, Rafael Bayarri-Olmos, Michael Fokuo Ofori, Lars Hviid, Peter Garred

**Affiliations:** ^1^Centre for Medical Parasitology, Department of Immunology and Microbiology, Faculty of Health and Medical Sciences, University of Copenhagen, Copenhagen, Denmark; ^2^Laboratory of Molecular Medicine, Department of Clinical Immunology, Rigshospitalet, Copenhagen, Denmark; ^3^Department of Immunology, Noguchi Memorial Institute of Medical Research, University of Ghana, Accra, Ghana; ^4^Centre for Medical Parasitology, Department of Infectious Diseases, Rigshospitalet, Copenhagen, Denmark

**Keywords:** malaria, complement, evasion, PfEMP1, antibodies, knobs

## Abstract

Members of the PfEMP1 protein family are expressed on the surface of *P. falciparum*-infected erythrocytes (IEs), where they contribute to the pathogenesis of malaria and are important targets of acquired immunity. Although the PfEMP1-specific antibody response is dominated by the opsonizing and complement-fixing subclasses IgG1 and IgG3, activation of the classical complement pathway by antibody-opsonized IEs does not appear to be a major immune effector mechanism. To study the molecular background for this, we used ELISA and flow cytometry to assess activation of the classical component pathway by recombinant and native PfEMP1 antigen opsonized by polyclonal and monoclonal PfEMP1-specific human IgG. Polyclonal IgG specific for VAR2CSA-type PfEMP1 purified from a pool of human immune plasma efficiently activated the classical complement pathway when bound to recombinant PfEMP1 in ELISA. In contrast, no activation of complement could be detected by flow cytometry when the same IgG preparation was used to opsonize IEs expressing the corresponding native PfEMP1 antigen. After engineering of a VAR2CSA-specific monoclonal antibody to facilitate its on-target hexamerization, complement activation was detectable in an ELISA optimized for uniform orientation of the immobilized antigen. In contrast, the antibody remained unable to activate complement when bound to native VAR2CSA on IEs. Our data suggest that the display of PfEMP1 proteins on IEs is optimized to prevent activation of the classical complement pathway, and thus represents a hitherto unappreciated parasite strategy to evade acquired immunity to malaria.

## Introduction

Malaria remains a major health problem with an estimated 219 million cases and 435,000 deaths in 2017 alone (1). The human disease is caused by several protozoan parasites of the genus *Plasmodium*, but *P. falciparum* is responsible for most severe cases and essentially all malaria mortality (2).

The particular virulence of *P. falciparum* is related to the unique ability of this parasite to express members of a family of clonally variant surface antigens called *P. falciparum* erythrocyte membrane protein 1 (PfEMP1) on the surface of the infected erythrocytes (IEs) (3). This enables sequestration of IEs in the microvasculature, mediated by interaction of PfEMP1 with vascular host receptors such as CD36, endothelial protein C receptor (EPCR), and oncofetal chondroitin sulfate (4–7). The ensuing accumulation of IEs in tissues can lead to severe disease, precipitated by excessive inflammation and circulatory dysfunction.

The PfEMP1 antigens are important targets of naturally acquired immunity to *P. falciparum* malaria, and semi-immune individuals living in areas of stable parasite transmission possess a broad repertoire of PfEMP1-specific antibodies, dominated by the opsonizing and complement-fixing subclasses IgG1 and IgG3 (8, 9). It is therefore noteworthy that activation of the classical complement pathway does not appear to play a major role in acquired immunity to IEs, although acquired immunity leads to activation of complement by antibody-opsonized sporozoites and merozoites (10, 11). Indeed, *P. falciparum* has evolved several mechanisms to evade activation of the alternative and classical pathways by hijacking soluble complement regulators to these developmental stages, emphasizing the clinical importance of complement-mediated attack on malaria parasites (12–16). The display of PfEMP1 on the IE surface is normally restricted to electron-dense protrusions known as “knobs” (17). Although knob-less variants can express PfEMP1 and thrive *in vitro*, knob expression is generally thought to be required for *P. falciparum* survival *in vivo*, and the protection from falciparum malaria afforded by several hemoglobinopathies is thought related to abnormal knob formation on IEs (18). Nevertheless, the role of knobs in *P. falciparum* survival remains unclear. In this article, we investigate the hypothesis that the knob restriction of PfEMP1 on the IE surface may have evolved to prevent classical complement activation by preventing on-target hexamerization of IgG (19). We show that although polyclonal, and even monoclonal, IgG can activate the classical complement pathway when bound to surface-bound recombinant PfEMP1, such activation does not occur when the IgG is bound to native PfEMP1 expressed on the IE surface.

## Materials and Methods

### Recombinant Protein Production

The full-length ectodomain of the VAR2CSA-type PfEMP1 IT4VAR04 (FV2) was produced in ExpiCHO-S cells as a recombinant C-terminal histidine-tagged protein, as described elsewhere (20).

A recombinant version of the human monoclonal IgG1 antibody (mAb) PAM1.4 (21), which is specific for a conformational epitope in VAR2CSA-type PfEMP1, was produced as described elsewhere by cloning and inserting the variable domains of the antibody into plasmids encoding the constant regions of the γ_1_-chain and the κ-chain (22). This antibody has previously been shown to bind both plastic-immobilized FV2 in ELISA and the corresponding native antigen on the IE surface (20).

We also produced two variants of this antibody (PAM1.4-E345K and PAM1.4-E430G) by introducing single-nucleotide substitutions in the γ_1_-chain, using the QuickChange site-directed mutagenesis kit (Agilent) according to manufacturer's instructions. Briefly, mutations were introduced by a PCR reaction of the entire plasmid with a high-fidelity DNA-polymerase and a complementary primer pair with the desired mutation (E345K forward: 5′-CCAAAGGGCAGCCCCGAAAACCACAGGTGTA-3′; E430G forward:5′-CCGTGATGCATGGGCTCTGCACAACCACT-3′; substitutions are underlined). Plasmids were subsequently sequenced to confirm the introduction of the substitutions. All the recombinant antibodies were produced in human embryonic kidney cells (293-F; Gibco) according to manufacturer's instructions. Briefly, the cells were grown to ~1.5 × 10^6^ cells/mL, and adjusted to 1 × 10^6^ cells/mL 1 h prior to transfection, and co-transfected with heavy- and light chain plasmids (0.5 μg DNA/plasmid/mL culture) and FreeStyle MAX reagent (1 μL/μg DNA). Cultures were incubated for seven days before harvesting the supernatant. The recombinant antibodies were purified using Protein G-coupled agarose beads (Pierce).

### IgG Purification From Human Plasma Samples

Total IgG was purified from pools of plasma from ten Ghanaian donors with natural exposure to *P. falciparum* parasites expressing VAR2CSA-type PfEMP1. The samples were collected in a previous study approved by the Institutional Review Board of Noguchi Memorial Institute for Medical Research, University of Ghana (study no. 038/10-11) (23). Samples were collected only after consent had been obtained in writing from each participant. One pool consisted of the ten available samples with the highest reactivities toward FV2, while the other consisted of the ten available samples with the lowest FV2 reactivities. IgG was purified from each pool of plasma using Gammabind G sepharose (GE Healthcare), using standard methodology. Affinity purification on FV2 was not employed, to allow direct comparison of the pools with high and low FV2 reactivity. However, previous studies have shown that PfEMP1 is the dominant IE target antigen of naturally acquired IgG (24).

### Classical Complement Pathway Activation ELISA

Flat-bottomed 96-well plates (Nunc) were coated (4°C, overnight in PBS) with FV2 (2 μg/mL), mAbs (PAM1.4; PAM1.4 E345K; PAM1.4 E430G), purified human IgG (Invitrogen), or human serum albumin (HSA; 10 μg/mL; Sigma Aldrich). Wells were blocked with PBS supplemented with TWEEN (0.05%) and BSA (1%) and subsequently incubated (1 h on shaker, as all subsequent steps) with the above-mentioned VAR2CSA-specific mAbs or with control reagents [mAb AB01, specific for a non-VAR2CSA-type PfEMP1 (25) or polyclonal rabbit anti-HSA (Dako)]. In some experiments, 96–well plates pre-coated with nickel, pre-blocked with BSA and coated with FV2 (5 μg/mL) in PBS supplemented with TWEEN (0.05%) were used (Pierce).

After incubation with antibody reagents, the plates were washed four times in Barbital-Tween buffer [sodium barbital (4 mM), NaCl (145 mM), CaCl_2_ (2.64 mM), MgCl_2_ (2.12 mM), Tween (0.05%)] and incubated (1 h, 37°C) in the same buffer supplemented with non-immune human serum (NHS; 1%) as source of complement components. After washing as above, bound complement components were detected with polyclonal rabbit anti-human C1q (2 μg/mL; Dako), rabbit anti-human C4c (1 μg/mL; Dako), rabbit anti-human C3c (1 μg/mL; Dako), or monoclonal mouse-anti human-terminal complement complex (TCC; 1 μg/L; clone ae11). All the complement-specific antibody reagents were biotinylated prior to the experiments. After the last washing as above, bound antibody was detected by incubation with streptavidin-conjugated horse radish peroxidase (1:2,000; GE Healthcare), followed by TMB ONE (ECO-TEK). The color reaction was terminated with H_2_SO_4_ (0.2 M), and quantified at 450 nm. Arbitrary units (AU) were calculated as (OD_Test_ - OD_Blank_) / (OD_Control_ - OD_Blank_).

### *P. falciparum* Culture *in vitro* and Selection for Expression of PfEMP1 and Knobs

The *P. falciparum* laboratory isolate IT4/FCR3 was cultured in serum-free medium as described elsewhere (26).

Parasites were selected by antibody panning and density separation monthly to ensure expression of the VAR2CSA-type PfEMP1 IT4VAR04 and IE surface knobs as described previously (27). Briefly, cultures with primarily late trophozoite-stage IEs were incubated (20 min, 37°C) in culture medium supplemented with gelatin (0.75%) to separate late trophozoite-stage knobby IEs from uninfected erythrocytes and ring-stage IEs. The late trophozoite-stage IEs were then incubated with DynaBeads A (DYNAL) that had been pre-incubated (30 min) with saturating amounts of PAM1.4 antibody. Bound IEs were isolated using a DynaMag (DYNAL).

### Classical Complement Activation on IEs

Late trophozoite-stage IEs were purified by magnet-activated cell sorting (Miltenyi Biotec) in PBS supplemented with BSA (1%) (28). The purified IEs (2 × 10^6^ cells/mL) were incubated (30 min, 4°C) with mAbs (10 μg/mL) or purified pooled human IgG (1 mg/mL), followed by incubation (1 h, 37°C) with NHS (1%) and compstatin (6 nM; Tocris) (to inhibit cleavage of C3). As positive and negative controls, type A and 0 erythrocytes were incubated with type 0 NHS and compstatin.

Complement components were detected by incubation (4°C, 30 min) with the same antibodies as above, but at different concentrations (anti-C1q: 30 μg/mL; anti- C4c: 0.5 μg/mL), followed by incubation with FITC-conjugated goat anti-rabbit IgG (1:150; Vector) and ethidium bromide (2 μg/mL; to visualize parasite DNA). Binding of human IgG to the IEs was detected in a similar way, using FITC-conjugated goat anti-human IgG (1:150; Jackson Immuno Research) (28). All incubations, dilutions, and washes were done in PBS supplemented with BSA (1%), except for the incubation with NHS and compstatin, which was done in BSA-supplemented VBS^++^ buffer containing Ca^2+^ and Mg^2+^ (Complement Technology). Samples were run on a Cytomics FC500 flow cytometer (Beckman Coulter). Single ethidium bromide-positive cells were analyzed for complement components and antibody labeling by FlowLogic (Inivai Technologies).

## Results

### Human Specific IgG Bound to Immobilized Recombinant PfEMP1 Activates the Classical Complement Pathway

**Figure 1:**
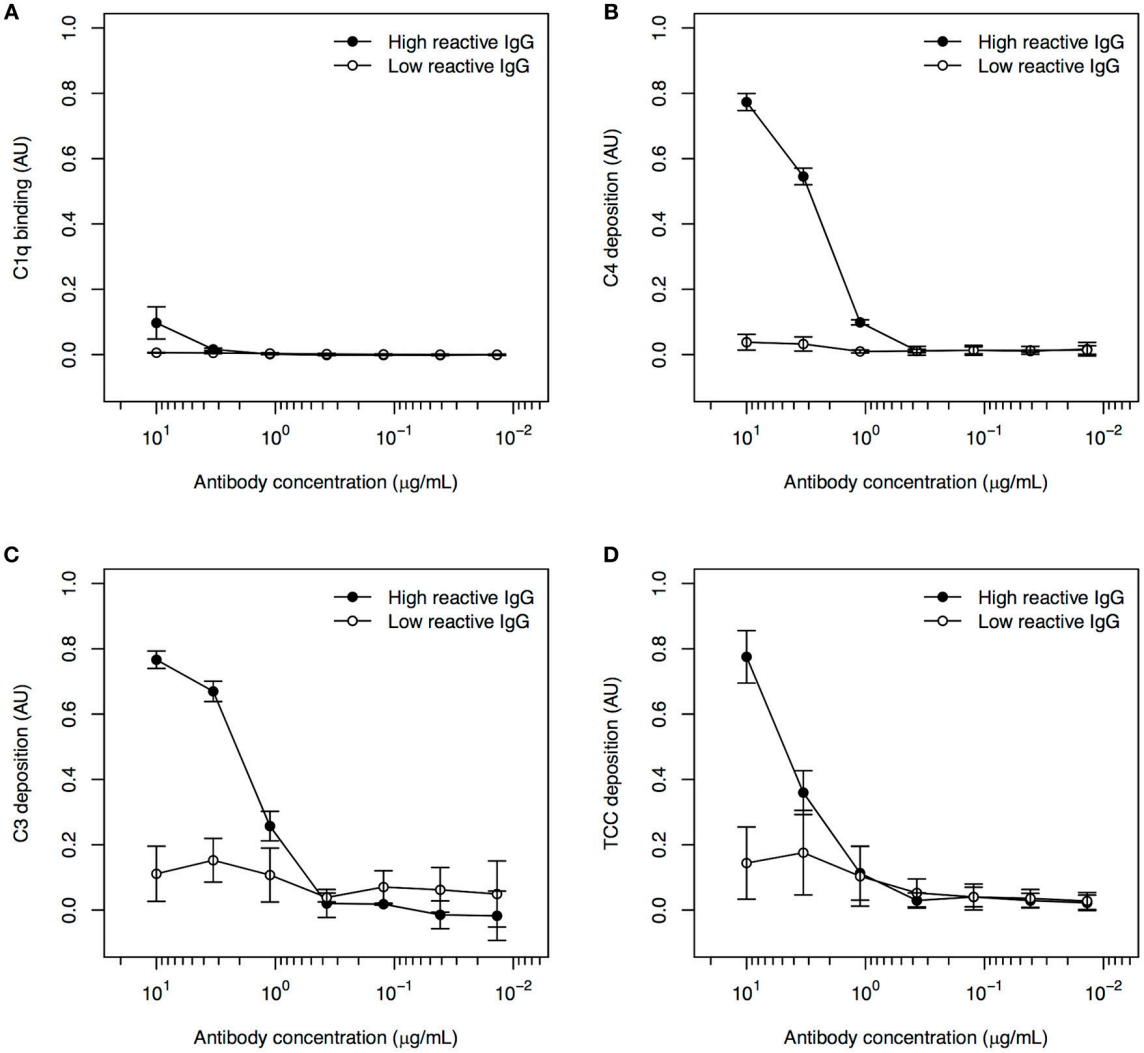
Classical complement pathway activation in ELISA, using IgG with high and low FV2-reactivity and FV2 coated directly to plastic. Binding of C1q **(A)**, deposition of C4 **(B)** and C3 **(C)**, and formation of TCC **(D)** to IgG purified from plasma pools with high (•) and low (°) FV2-reactivity and bound to FV2 immobilized directly on plastic. All data were normalized relative to control wells coated with HSA and incubated with rabbit anti-HSA. Data points representing the means and standard deviations (error bars) of three individual experiment are shown.

To our knowledge, the ability of PfEMP1-specific human IgG to activate the classical complement pathway has not been reported previously. Binding of C1q (Figure 1A), deposition of C4 (Figure 1B) and C3 (Figure 1C), and formation of TCC (Figure 1D) were detected in recombinant FV2-ELISA after incubation with human IgG purified from a plasma pool with known high FV2-reactivity, followed by NHS as a source of complement components. When IgG from the pool of low-reactive plasma was used instead of the highly FV2-reactive IgG, no complement deposition was detected (Figure 1). We conclude that naturally acquired PfEMP1-reactive IgG is able to activate the classical complement pathway when bound to recombinant FV2 that has been randomly immobilized on plastic.

### Human Specific IgG Bound to Native PfEMP1 on Infected Erythrocytes Does Not Activate the Classical Complement Pathway

**Figure 2:**
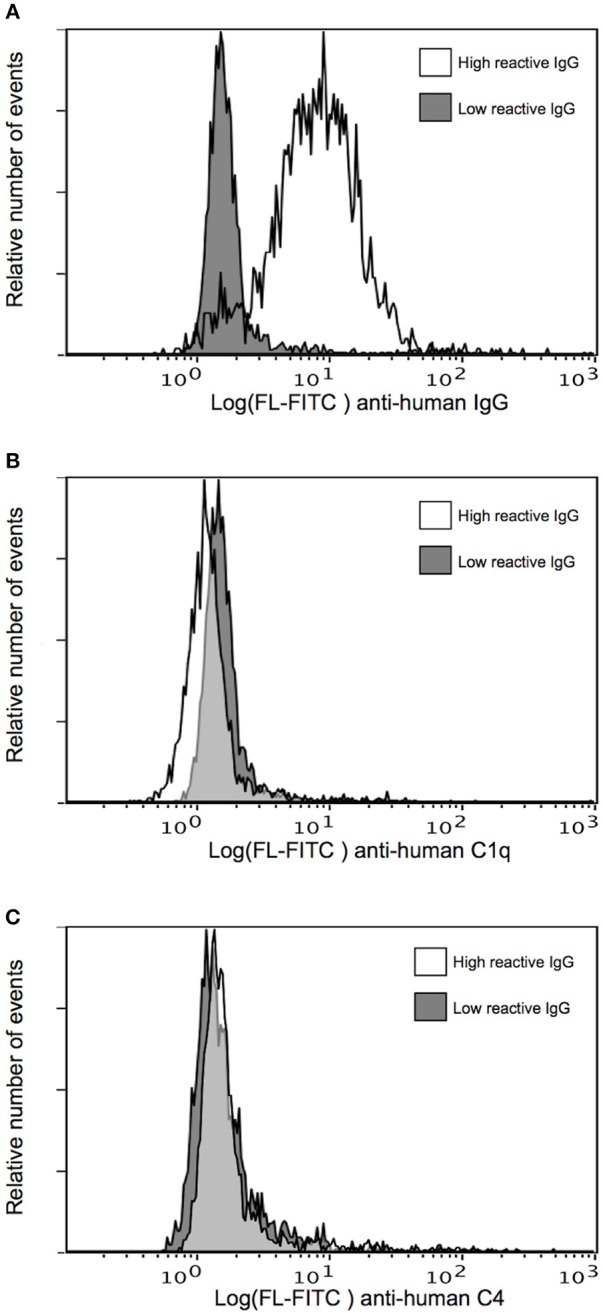
Classical complement pathway activation on IEs, using IgG with high and low FV2 reactivity. Binding of purified immune IgG **(A)** and C1q **(B)**, and deposition of C4 **(C)** to *P. falciparum*-infected erythrocytes selected *in vitro* to express the VAR2CSA-type PfEMP1 IT4VAR04. Representative results of two independent experiments using IgG with high (white) and low (gray) FV2-reactivity are shown.

We next assessed the ability of human IgG purified from the same plasma pools to activate complement when bound to IEs expressing the native PfEMP1 (IT4VAR04) represented by FV2. IgG purified from the highly FV2-reactive pool efficiently labeled the IEs, in contrast to the low FV2-reactivity IgG (Figure 2A). However, essentially no binding of C1q (Figure 2B) or deposition of C4 (Figure 2C) could be detected after incubation of IEs with either IgG preparation. The functionality of the assay was confirmed in experiments using uninfected type A and type 0 erythrocytes incubated with type 0 NHS (Figure S1). These findings suggest that the distribution of PfEMP1 on IEs inhibits classical complement activation, possibly due to the clustered, knob-restricted distribution of the antigen.

### Activation of Complement by Monoclonal IgG Bound to Immobilized Recombinant PfEMP1 Depends on Antigen Orientation

It was recently reported that complement activation by IgG requires on-target, Fc-dependent hexamerization of the antibody (19). We therefore proceeded to test whether the lack of complement activation by PfEMP1-specific IgG bound to IEs was due to an inability of the antibodies to form hexamers after binding to native PfEMP1 on the IE surface. To do so, we used IEs selected for surface expression of IT4VAR04, and a mAb (PAM1.4) with specificity for VAR2CSA-type PfEMP1 (including IT4VAR04). We also used two variants of this mAb (PAM1.4-E345K and PAM1.4-E430G), where we had introduced mutations in the Fc-region (E345K and E430G, respectively), known to enhance on-target hexamerization of IgG (19, 29).

**Figure 3:**
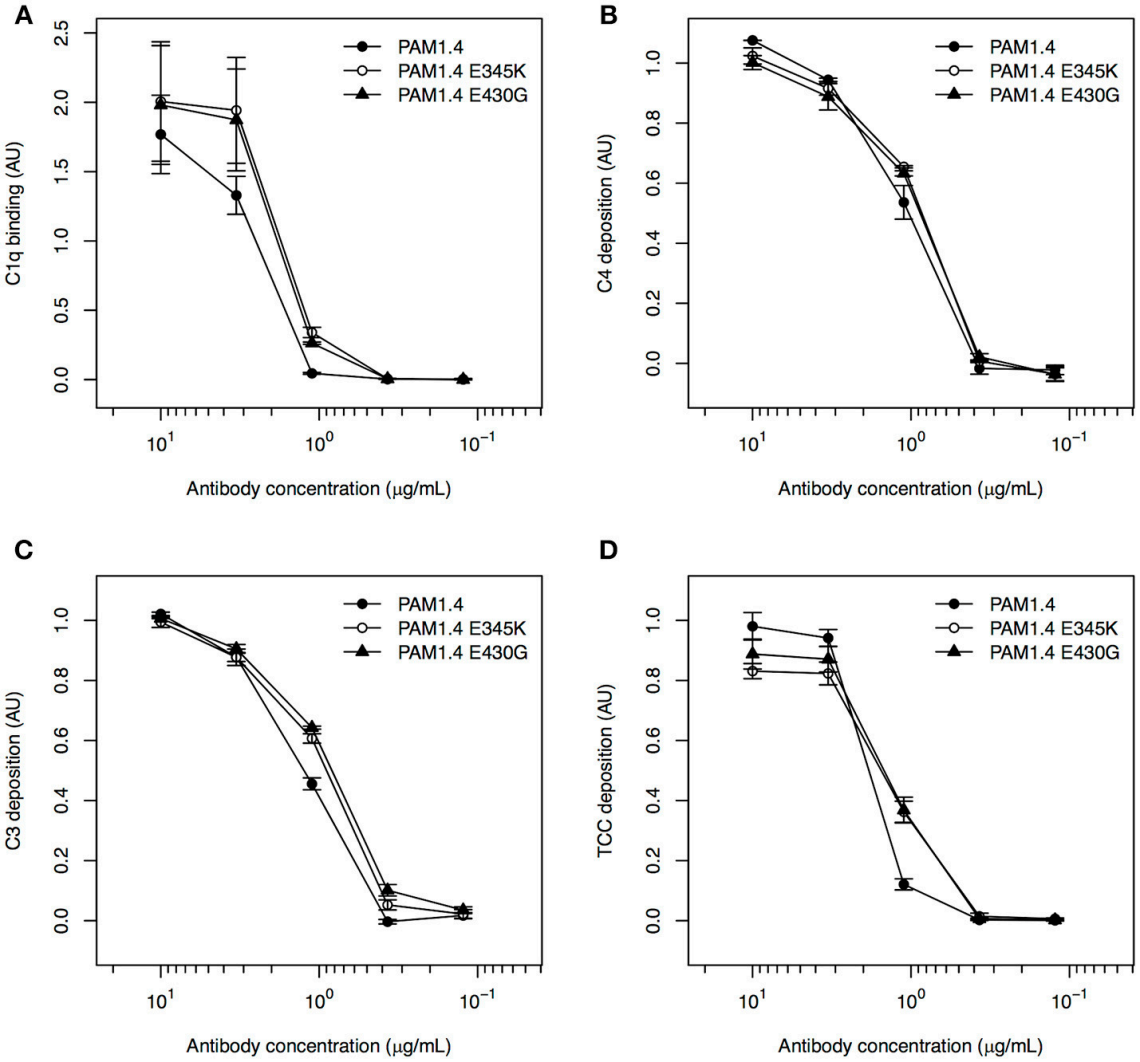
Classical complement pathway activation in ELISA, using FV2-specific monoclonal IgG with and without mutations enhancing on-target hexamerization, coated directly to plastic. Binding of C1q **(A)**, deposition of C4 **(B)** and C3 **(C)**, and formation of TCC **(D)**, using the FV2-specific mAbs PAM1.4 (•), PAM1.4-E345K (°), and PAM1.4-E430G (▴) immobilized directly on plastic. Data representation as in Figure 1.

All three mAbs had similar ability to activate complement when coated directly to ELISA plates, as binding of C1q (Figure 3A), as well as deposition of C4 (Figure 3B), C3 (Figure 3C), and TCC (Figure 3D) could be detected in a concentration-dependent manner. This agrees with the observation that enhanced complement activation by hexamerization-improved Fc mutants requires binding of the antibodies to their cognate antigen (29). To confirm this requirement directly, the mAbs were next applied in a setup where the ELISA plates were first coated with FV2 as in the experiments with purified immune IgG above. However, neither PAM1.4 nor the Fc-mutated variants of the mAb activated complement in this setup (Figure S2).

**Figure 4:**
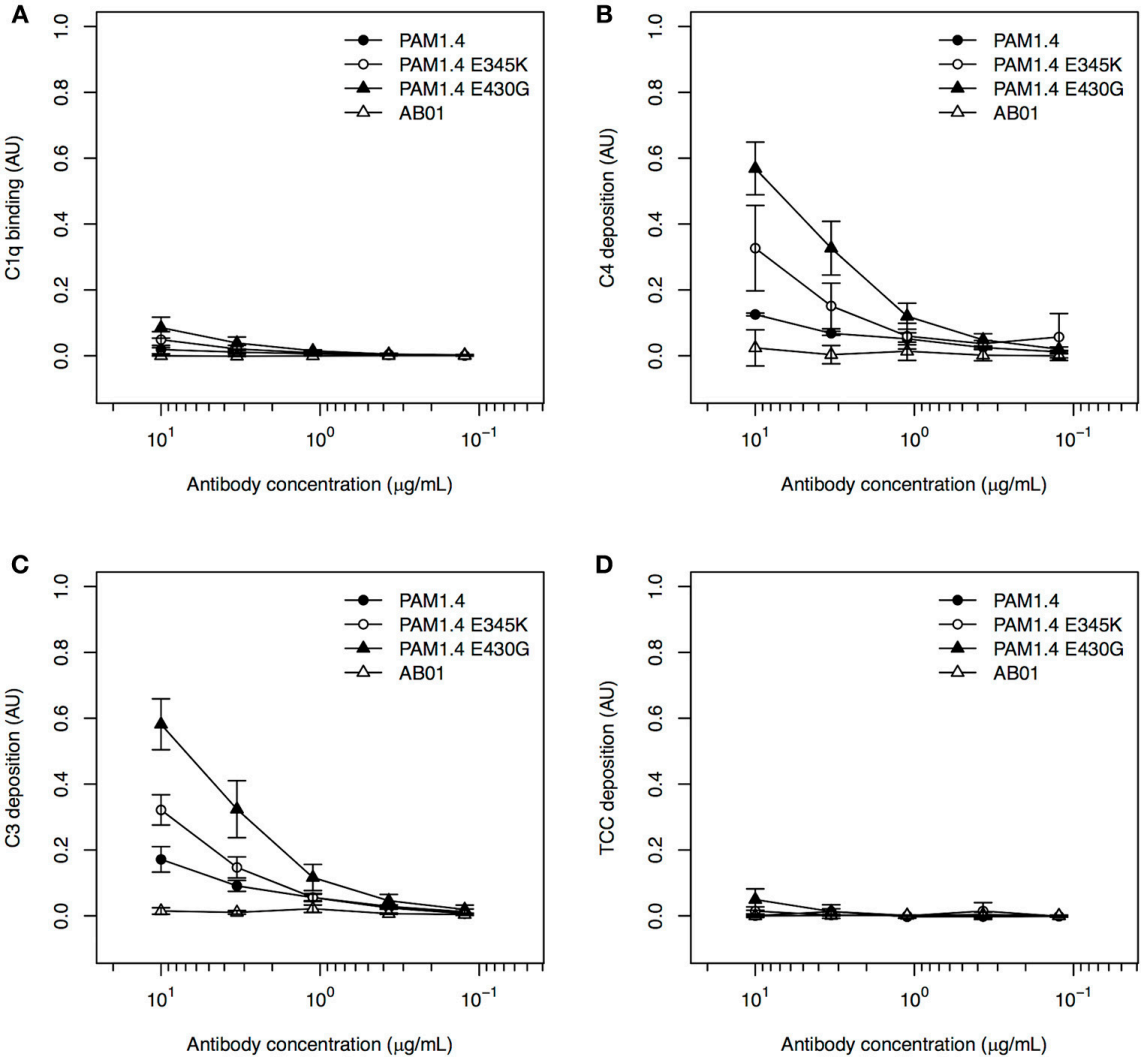
Classical complement pathway activation in ELISA, using FV2-specific monoclonal IgG, bound to plastic coated with FV2 by His-tag::nickel interaction. Binding of C1q **(A)**, deposition of C4 **(B)** and C3 **(C)**, and formation of TCC **(D)** on the FV2-specific mAbs PAM1.4 (•), PAM1.4-E345K (°), and PAM1.4-E430G (▴), and the control mAb AB01 (Δ) in assays employing uniformly oriented FV2 immobilized by interaction between the C-terminal His-tag on FV2 and nickel-coated plastic. Data representation as in Figure 1.

In the studies first identifying the complement activation-enhancing mutations, the assays involved cell lines over-expressing the targeted antigen (19, 29). We therefore speculated that the lack of complement activation in our setup with recombinant FV2 bound to plastic might be related to the random orientation of the immobilized antigen, in contrast to the usually uniform display of a given antigen on the surface of cells. To approximate such an ordered display, we exploited the fact that our FV2 protein has a C-terminal poly-histidine tag, and immobilized the recombinant protein on ELISA plates pre-coated with nickel. Because the nickel ions bind to the FV2 poly-histidine tags, this should facilitate homogeneous orientation of the antigen similar to the orientation of the native protein on IEs, although the distribution of the FV2 would remain more homogenous than the knob-restricted distribution of native PfEMP1 proteins on IEs. In this setup, binding of C1q (Figure 4A), and in particular deposition of C4 (Figure 4B) and C3 (Figure 4C), could be detected in a concentration-dependent manner for all three mAbs, whereas TCC formation was minimal (Figure 4D). Although this might theoretically be due to activation of the alternative pathway by binding of mannan-binding lectin to glycosylation determinants on the recombinant antibodies, this appears unlikely. Firstly, the glycosylation pattern of the cells used to express the recombinant antibodies is very similar to native human IgG (30). Secondly, the hexamerization-enhanced Fc-region mutants, in particular PAM1.4-E430G, were superior to PAM1.4 (if the binding of C4 and C3 were due to activation of the lectin rather than the classical pathway, similar activation by wildtype PAM1.4 and the two hexamerization-enhanced mutants would be expected). When this improved assay was used to test complement activation by purified immune IgG, C4 deposition could be detected with both the high and the low FV2-reactive preparation, although the highly FV2-reactive IgG was superior (Figure S3).

Our data confirm that the E345K and E430G mutations in the Fc-region enhance the ability of IgG to activate complement, probably by facilitating on-target hexamerization. Furthermore, the results highlight antigen orientation as an important parameter in *in vitro* assays of classical complement pathway activation.

### Hexamerization-Enhancing Fc Mutations Do Not Lead to Activation of Complement by Monoclonal PfEMP1-Specific IgG Bound to Infected Erythrocytes

**Figure 5:**
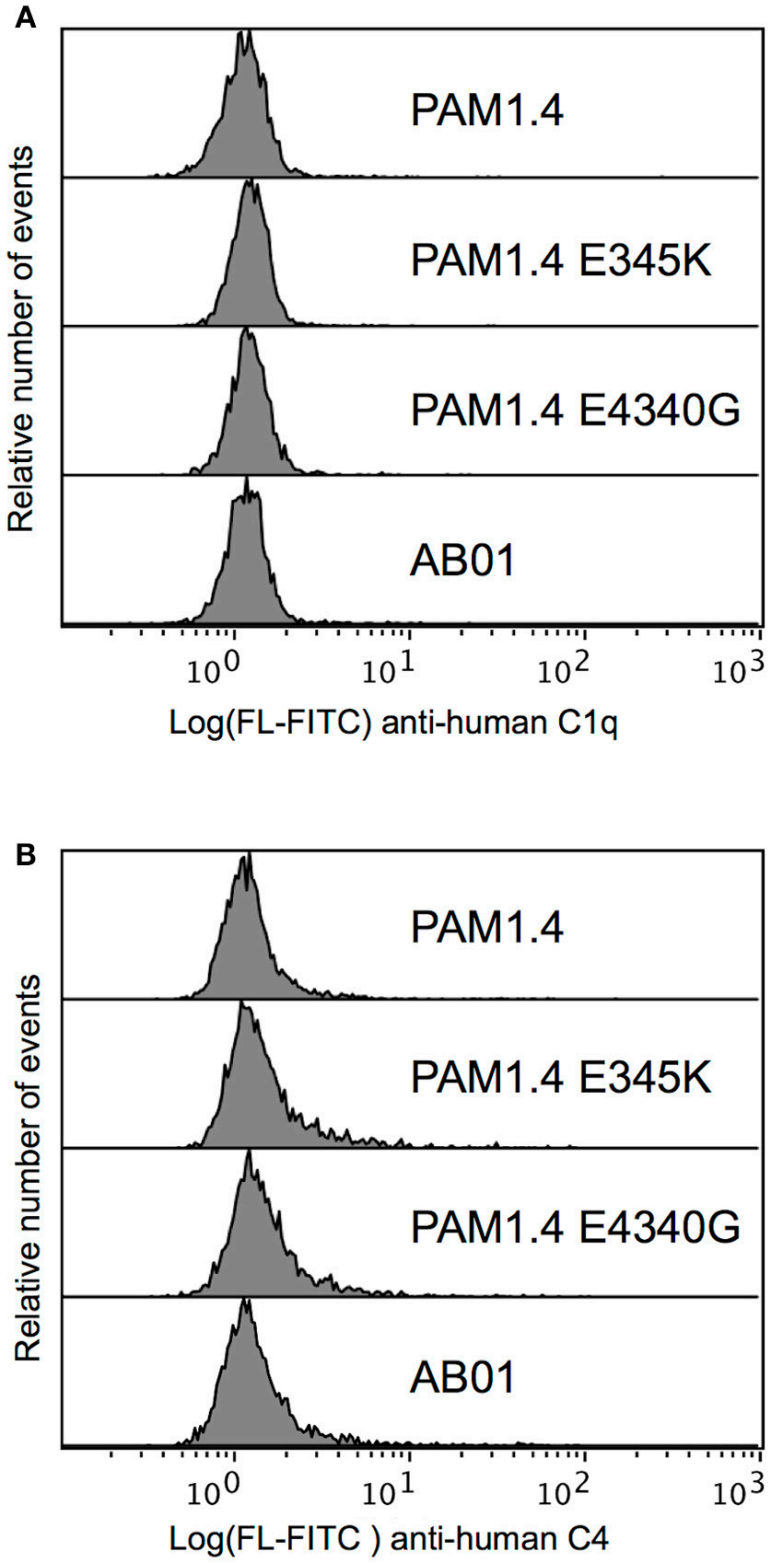
Classical complement pathway activation on IEs, using FV2-specific monoclonal IgG with and without mutations enhancing on-target hexamerization. Binding of C1q **(A)** and deposition of C4 **(B)**, using the FV2-specific mAbs PAM1.4 (Top), PAM1.4-E345K (Second), and PAM1.4-E430G (Third), or the control mAb AB01 (Bottom). Representative results of two independent experiments are shown.

To investigate whether the lack of complement activation on IEs (Figure 2) could be overcome by enhancing the antibody capacity for on-target hexamerization, we tested the ability of PAM1.4 and the two Fc mutants to activate complement when bound to IEs. We did not detect C1q binding (Figure 5A) or C4 deposition (Figure 5B) with any of the mAbs. It thus appears that the distribution of native PfEMP1 prevents hexamerization of monoclonal IgG in a way that could not be overcome by Fc mutations enhancing the ability of PAM1.4 to form hexamers. Although we cannot exclude that other mAbs might be able to form hexamers when bound to PfEMP1 on the IE surface, or that on-target hexamerization might occur with Fc-mutated polyclonal IgG preparations, it seems most likely that PfEMP1 is distributed on the IE surface in a way that prevents the interactions among Fc regions of adjacent IgG molecules that would facilitate binding of C1q and activation of the classical complement pathway.

## Discussion

IgG antibodies specific for the asexual parasites multiplying in the blood are a key element in naturally acquired protective immunity to *P. falciparum* malaria (31, 32). Antibodies to antigens on the surface of the IEs – in particular PfEMP1 – are of particular importance in this respect (3). This is probably due to their ability to block the vascular sequestration of IEs, which can otherwise cause inflammation and circulatory dysfunction (33). Unsurprisingly, *P. falciparum* has evolved a range of strategies to avoid PfEMP1-specific immunity, such as antigen polymorphism, clonal antigenic variation, acquisition of soluble host factors etc. (34).

The antibody response to *P. falciparum* asexual blood stage antigens, including the VAR2CSA-type PfEMP1 studied here, is dominated by IgG1 and IgG3 (35). It is therefore likely that phagocytosis of merozoites and IEs opsonized by antibody and complement also contribute significantly to parasite clearance. Although the role of the complement system in *P. falciparum* infections has been the focus of several recent studies, most have focused on the alternative pathway. The ability of IEs (13), merozoites (14), and gametocytes (12) to acquire the soluble complement regulator Factor H to their surface thus clearly suggests that this activation pathway is important in controlling parasitemia, and likely has forced the parasites to evolve strategies to evade this host defense. A very recent study indicates that this host-parasite tug-of-war is even more complicated, and that Factor H-related protein 1 may be involved in a host effort to overcome malaria parasite evasion of complement attack by acquisition of Factor H (36).

The clinical importance of classical complement pathway activation following opsonization by IgG has been less studied, although binding of C1q to IgG-coated merozoites and sporozoites has been associated with protection of malaria in patients from Oceania and Africa (10, 11). In addition, evasion of such immunity by hijacking of C1-inhibitor by merozoites was recently reported (15). The clinical relevance of antibody-dependent complement attack on merozoites may be limited by the fact that this free-living stage is only exposed to antibody and complement for 1-2 min before invading a new erythrocyte. It is questionable whether this is long enough for antibody binding and classical complement attack, as *in vitro* experiments required >2.5 min. before C3 deposition could be demonstrated (15). By then, many merozoites would be expected to have safely reinvaded (37). In the case of sporozoites, the parasite is exposed to antibody and complement for longer (38), and it therefore seems more likely that complement plays a decisive role in the immune response against this developmental stage. However, the intra-erythrocytic parasites should theoretically be vulnerable to classical complement attack for the ~30 of the 48-h asexual life cycle, where they express PfEMP1 on the IE surface to facilitate tissue sequestration and avoidance of destruction in the spleen (39). This notwithstanding, little is known about classical complement attack on IEs, let alone parasite strategies to evade this threat.

Here, we show that human IgG purified from plasma and having high reactivity to PfEMP1 can activate the classical complement pathway in ELISA. However, no complement activation was seen when the same IgG was bound to the corresponding native PfEMP1 on the surface of IEs. The molecular dimensions of IgG and PfEMP1 molecules, combined with estimates of the number of PfEMP1 molecules per IE (40–46) suggest that on-target hexamerization [required for efficient activation of the classical complement cascade by IgG 19] would occur if the PfEMP1 molecules were evenly distributed over the IE surface. We therefore hypothesized that the lack of activation is related to the knob-restricted expression of PfEMP1 on the IE surface, which prevents on-target hexamerization of IgG molecules bound to PfEMP1 molecules on neighboring knobs, as these are too far apart. The clustered distribution might thus represent a hitherto unidentified strategy by *P. falciparum* to evade acquired, IgG-mediated protective immunity. However, the molecular dimensions of knobs make on-target hexamerization of IgG bound to different PfEMP1 molecules within a given knob theoretically possible. We therefore produced two PfEMP1-specific mAbs with substitutions in the Fc-region that enhance their capacity for on-target hexamerization and complement activation (19, 29). Although we could demonstrate enhanced complement-activating capacity of Fc-mutated mAbs by ELISA, the mutations did not suffice to activate complement following binding of the mAbs to native PfEMP1 on the IE surface. Even with polyclonal immune IgG containing IgG with specificity for the many antibody epitopes that exist in VAR2CSA (21), we did not find evidence of activation of the classical complement cascade at the IE surface. Although the reason for the above observations is not known, the simplest explanation is that PfEMP1 molecules are not evenly distributed, even within the confines of a single knob, but are instead clustered together. Whether this is the case is not currently known, however.

Erythrocytes are inherently susceptible to complement attack, and they therefore possess endogenous membrane-bound complement regulators such as decay-accelerating factor (CD55) and protectin (CD59) to prevent inadvertent phagocytosis and lysis of complement-opsonized erythrocytes. Although CD59 has been reported as the factor preventing complement-mediated lysis of IEs (47), IE lysis is likely to be less important than opsonization for phagocytosis. In this study, we decided to focus on complement components upstream of the erythrocyte membrane-bound complement regulators' point of action, to avoid complications imposed by the need to enzymatically remove membrane-bound regulators.

To conclude, we report that although PfEMP1-specific IgG can activate the classical complement pathway in a system where the antigens are homogeneously distributed, this appears not to happen at the IE surface, where PfEMP1 display is restricted to well-defined knobs. The most parsimonious explanation for this discrepancy is that the focal display of native PfEMP1 interferes with the on-target hexamerization of IgG, which is a requirement for binding of C1q and activation of the classical complement cascade. The knob-restricted display may thus represent a hitherto unrealized strategy of *P. falciparum* to evade acquired protective immunity.

## Author Contributions

ML, LH, and PG formulated the hypothesis and designed the experiments and analyzed the data and wrote the paper. ML, MQ, SD, and RB-O produced the recombinant proteins. ML carried out all the experiments. MO was responsible for collection of the biological samples. All authors reviewed and edited the manuscript.

### Conflict of Interest Statement

The authors declare that the research was conducted in the absence of any commercial or financial relationships that could be construed as a potential conflict of interest.

## Abbreviations

AU: arbitrary units
BSA: bovine serum albumin
FV2: full-length ectodomain of the VAR2CSA-type PfEMP1 IT4VAR04
HAS: human serum albumin
IE: infected erythrocyte
mAb: monoclonal antibody
NHS: non-immune human serum
PfEMP1: *Plasmodium falciparum* erythrocyte membrane protein-1
TCC: terminal complement complex.
