# Supplementary material for: Evasion of Classical Complement Pathway Activation on Plasmodium falciparum-Infected Erythrocytes Opsonized by PfEMP1-Specific IgG
Source: Front Immunol. 2019 Jan 7;9:3088. doi: 10.3389/fimmu.2018.03088 (PMC6330326; doi:10.3389/fimmu.2018.03088)
Supplement: Supplementary file 1 [file Data_Sheet_1.PDF]

Supplementary Material

Evasion of classical complement pathway activation on *Plasmodium falciparum*-infected erythrocytes opsonized by PfEMP1-specific IgG

Mads Delbo Larsen, Maria del Pilar Quintana, Sisse Bolm Ditlev, Rafael Bayarri-Olmos, Michael Fokuo Ofori, Lars Hviid* and Peter Garred*

*** Correspondence:** Peter Garred: peter.garred@regionh.dk and Lars Hviid: lhviid@sund.ku.dk

1 Supplementary figures

1.1 Figure S1

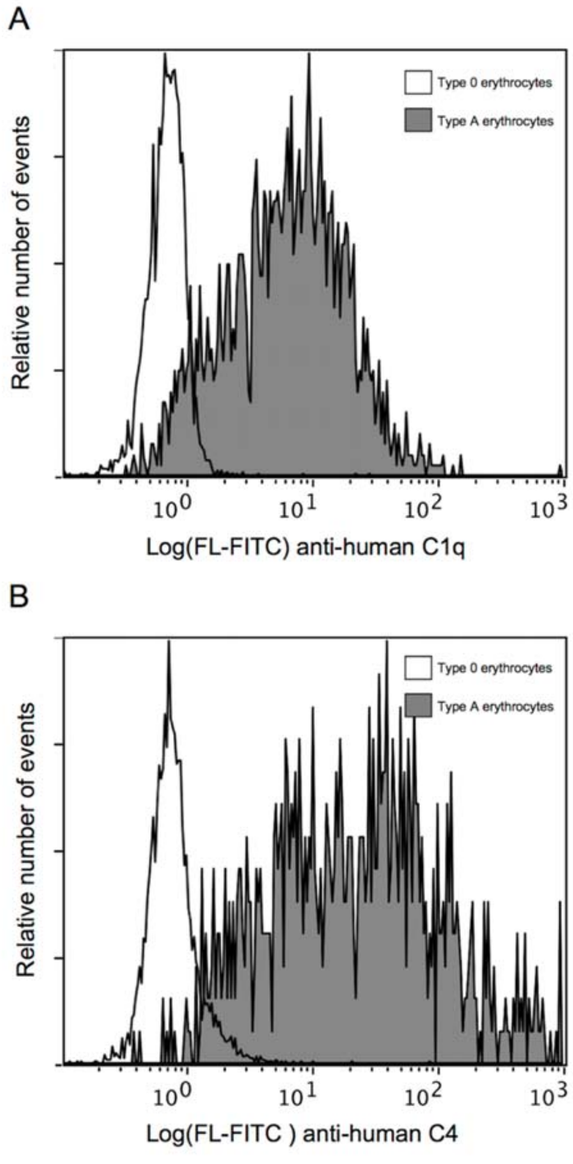

1.2 Figure S2

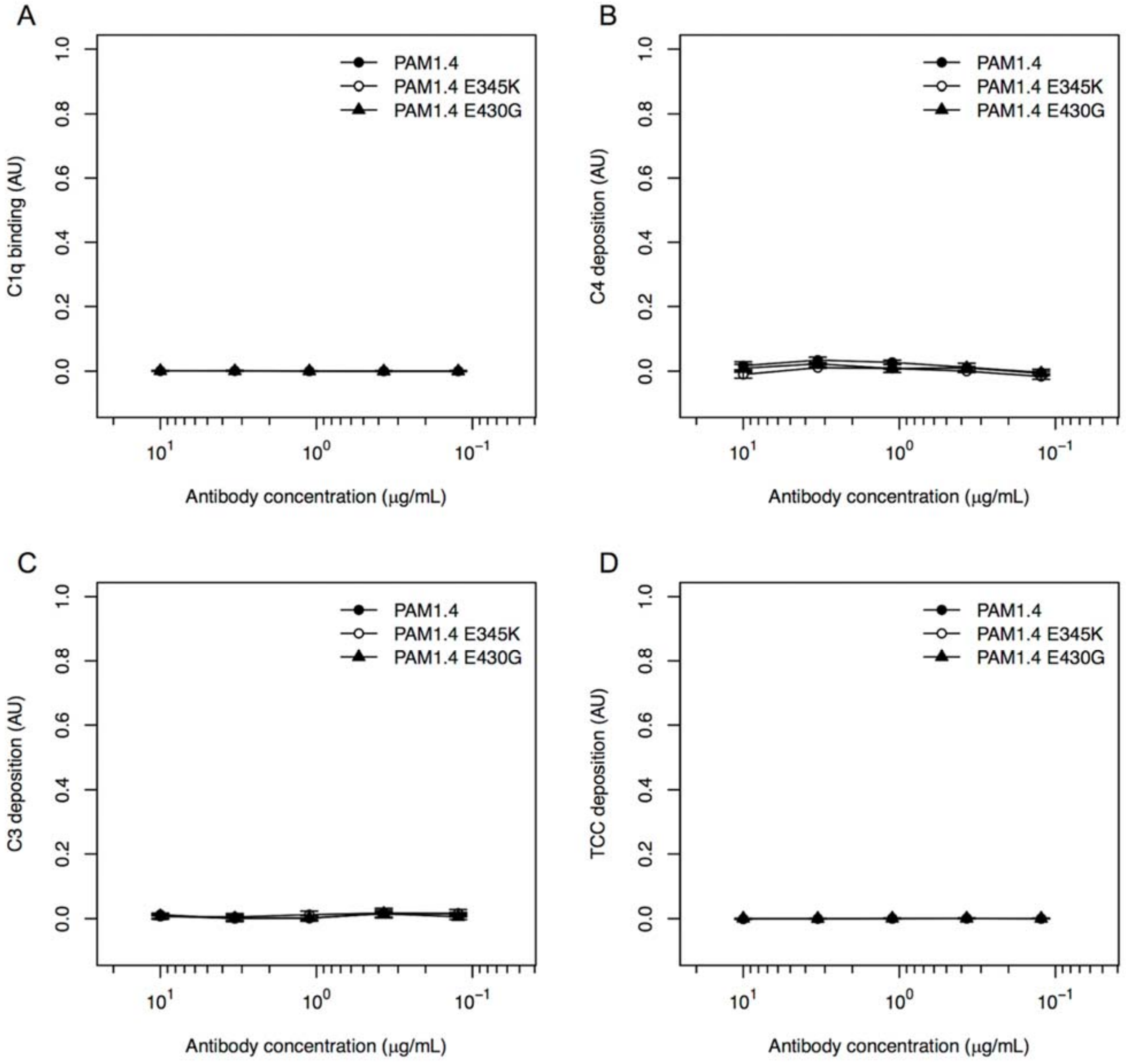
A
B
C
D
PAM1.4
PAM1.4 E345K
PAM1.4 E430G
C1q binding (AU)
C4 deposition (AU)
C3 deposition (AU)
TCC deposition (AU)
Antibody concentration (μg/mL)

1.3 Figure S3

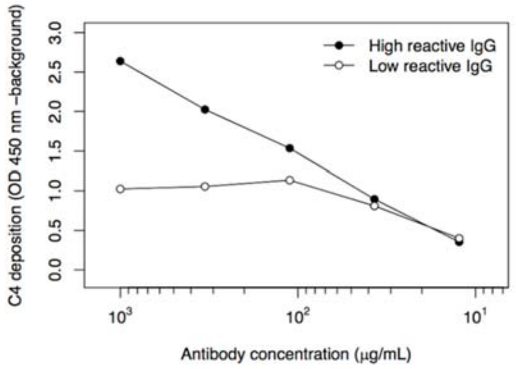

2 Supplementary figure legends

2.1 Figure S1 Classical complement pathway activation on type A and 0 erythrocytes by type 0 NHS

Binding of C1q (**A**) and deposition of C4 (**B**) to type A (gray) and type 0 (white) erythrocytes incubated with type 0 serum. Representative results of two independent experiments are shown.

2.2 Figure S2 Classical complement pathway activation in ELISA, using FV2-specific monoclonal IgG with and without mutations enhancing on-target hexamerization and FV2 coated directly to plastic.

Binding of C1q (**A**), deposition of C4 (**B**) and C3 (**C**), and formation of TCC (**D**), using the FV2-specific mAbs PAM1.4 (●), PAM1.4-E345K (○), and PAM1.4-E430G (▲) bound to immobilized FV2 coated directly on plastic. Data representation as Fig. 1

2.3 Figure S3 Classical complement pathway activation in ELISA, using IgG with high and low FV2-reactivity, bound to plastic coated with FV2 by His-tag::nickel interaction

Deposition of C4 by high FV2-reactive IgG (●) and low FV2-reactive IgG (○), in assays employing uniformly oriented FV2 immobilized by interaction between the C-terminal His-tag on FV2 and nickel-coated plastic.
